# The Preparation of Porous Sol-Gel Silica with Metal Organic Framework MIL-101(Cr) by Microwave-Assisted Hydrothermal Method for Adsorption Chillers

**DOI:** 10.3390/ma10060610

**Published:** 2017-06-02

**Authors:** Kasimayan Uma, Guan-Ting Pan, Thomas C.-K. Yang

**Affiliations:** 1 Centre for Precision Analysis and Research Center, National Taipei University of Technology, Taipei 106, Taiwan; 2 Department of Chemical Engineering, National Taipei University of Technology, Taipei 106, Taiwan; t6679013@gmail.com

**Keywords:** microwave-assisted hydrothermal technique, sol-gel method, metal organic framework composites, adsorbent

## Abstract

Metal organic framework (MOF) of MIL-101(Cr)-Silica (SiO_2_) composites with highly mesoporous and uniform dispersions were synthesized by a microwave-assisted hydrothermal method followed by the sol-gel technique. Water vapor adsorption experiments were conducted on the MIL-101(Cr)-SiO_2_ composites for industrial adsorption chiller applications. The effects of MIL-101(Cr)-SiO_2_ mixing ratios (ranging from 0% to 52%), the surface area and amount of Lewis and Brønsted sites were comprehensively determined through water vapor adsorption experiments and the adsorption mechanism is also explained. The BET and Langmuir results indicate that the adsorption isotherms associated with the various MIL-101(Cr)-SiO_2_ ratios demonstrated Type I and IV adsorption behavior, due to the mesoporous structure of the MIL-101(Cr)-SiO_2_. It was observed that the increase in the amount of Lewis and Brønsted sites on the MIL-101(Cr)-SiO_2_ composites significantly improves the water vapor adsorption efficiency, for greater stability during the water vapor adsorption experiments.

## 1. Introduction

Recently, remarkable progress has been made in the use of porous materials as functional components in a variety of gas storage/separation, sensing, catalysis and proton conduction applications [1]. MOF materials are interesting for industrial applications due to their high surface area, special crystalline structure, tunable pore size, and other unique properties [2]. However, as reported by Li and Zeng, several important drawbacks remain with these MOFs such as low chemical stability and weak mechanical strength [3], which limit their usage in industrial applications. Nevertheless, the physical and chemical properties of the MOF composites have showed a great deal of interest for adsorption applications. MOF-based composites can be prepared by the physical or chemical integration of a basic MOF material into an inorganic or polymeric substrate [4,5]. For example W. Martin et al. [6] reported on the thermal and chemical stability of MIL-100 (Fe, Cr) and MIL-101(Cr) MOFs in water vapor adsorption studies. They found the MOFs of MIL-100(Fe, Cr) and MIL-101(Cr) to be promising candidates for the development of adsorption chillers, with the adsorption values reaching to 0.79 g/g (i.e., 43.88 mM/g). In addition, as reported by Ferey et al. [7], materials with high micro-pore volumes and large surface areas are desirable for adsorption applications because they allow faster mass diffusion and are capable of hosting large molecules. However, most microporous materials have diffusion limits, which affect the diffusion rate of the composite and the resultant chemical reactions [8]. Recently, some research groups have reported on the synthesis of MOF composite thin films on bare and modified nonporous silica and alumina surfaces by microwave-assisted and solvothermal methods [9,10]. However, it is difficult to synthesize perfect MOF composites because the uniformity of the dispersion is ruined by crystallization and agglomeration of the powder. It is important to modify the MOF with mesoporous silica so as to increase the adsorption ability and the number of specific adsorption sites, in order to solve the diffusion problem. Moreover, the coexistence of nano-sized silica and MOFs with highly dispersed microstructures around the silica matrix may be achieved during the synthesis process. The aspect ratios, MOF nanostructure, surface area and pore volume represent additional constraints that need to be considered in order to improve the integration between SiO_2_ and MOF in a composite material. In recent past, some of the works have been reported on the MOF-SiO_2_ composites to enhance the adsorption/separation behaviors of MOF [11,12,13,14].

MOF materials have been synthesized by various methods, including microwave-assisted hydrothermal [15,16], hydrothermal [17,18], electrochemical [19,20], sonochemical [21], mechanochemical [22,23,24], ionothermal [25,26,27,28], drygel conversion [29,30], and microfluidic [31] approaches. Among these fabrication techniques, microwave-assisted hydrothermal methods offer a number of advantages other than synthetic routes, particularly in the case of MOFs [10]. Also, a sol-gel approach is the most facile route for the production of highly-ordered and controlled patterns and architectures, with an improved production rate and functionality [32]. A few studies have also been devoted to exploring the mechanism of water vapor adsorption in MOF materials [1,6,33,34]. Many researchers have studied increasing the mesoporosity in MOF material, but it has been found that small changes in the molar ratios of the reactants or solvents can result in nanoporous materials with poor crystallinity. 

In this study, we demonstrate a facile approach for the synthesis of MIL-101(Cr)-SiO_2_ composites, utilizing the microwave-assisted hydrothermal and sol-gel methods. We present a bottom-up synthesis strategy which produces a good aspect ratio of MIL-101(Cr)-SiO_2_ composites. The MIL-101(Cr) ratio and good dispersion in the silica matrix lead to good performance. Further, we evaluate the physical and chemical properties of the MIL-101(Cr)-SiO_2_ composites on the role of water-vapor adsorbents for applications in adsorption chillers.

## 2. Results and Discussion

In this work, we prepared pristine MIL-101(Cr) samples using a microwave-assisted, hydrothermal method, with specific reaction times (e.g., 20, 30, 40, 50 and 60 min). There was no change in the size of the MIL-101(Cr) crystals obtained, even after increasing the reaction time to 60 min. In order to obtain a good crystalline structure we used different ratios of chromium nitrate/terephthalic acid/hydrofluoric acid (1:1:4, 1:1:2 and 1:1:1.4) as seeds for growth of the MIL-101(Cr)-SiO_2_ composites. The SiO_2_ modified MIL-101(Cr) composites were characterized with different analytical methods to study the composition and structure of the MIL-101(Cr)-SiO_2_ composites.

### 2.1. FTIR Spectral Analysis

**Figure 1:**
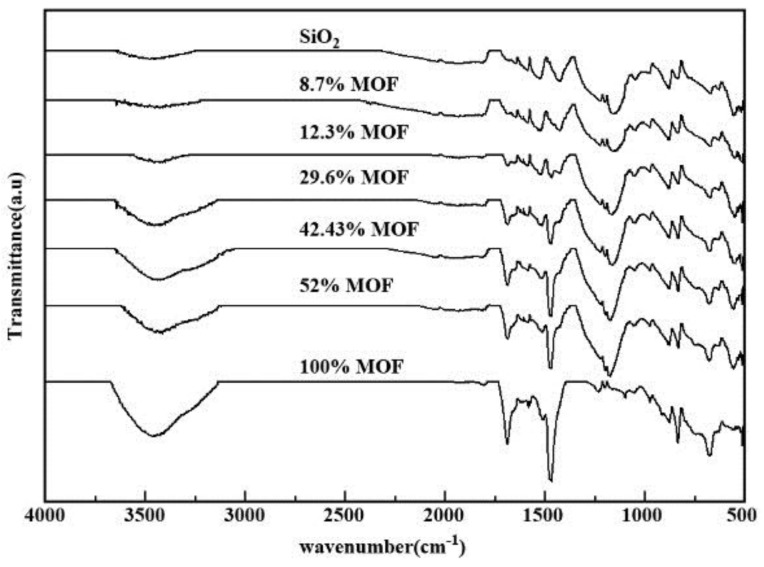
FTIR spectra of MIL-101(Cr) (n%)-SiO_2_ composites with different MIL-101(Cr) percentages.

Figure 1 shows the FTIR spectra of the as-prepared MIL-101(Cr)-n%)-Si samples with different MIL-101(Cr) percentages. The broad absorption band, which appears at 3500 cm^−1^ corresponds to the OH stretching vibration derived from the strongly H-bonded COOH groups [35]. In addition, the peaks at 1605 cm^−1^ and 1425 cm^−1^ correspond to the asymmetric deformation modes of the ammonia species (NH^4+^) absorbed on the Lewis sites and Brønsted sites, respectively [36]. The peaks observed in the range between 1300 cm^−1^ and 1750 cm^−1^ confirm the presence of dicarboxylate (νas(COO), νs(COO), and ν(C-C) vibrations) in the structure of the metal organic framework [37]. The bands at 1635, 1117 and 749 cm^−1^ can be attributed to the vibrations of the aromatic rings (σ(C-H) and γ(C-H)) and C=O group [38], respectively. In addition, the peaks at 800, 972 and 1092 cm^−1^, indicate stretching and the asymmetric vibration of the Si-O-Si [39], whereas the absorption peak at 577 cm^−1^ indicates Cr–O vibration in the metal organic framework [40]. 

### 2.2. Powder XRD Measurements

**Figure 2:**
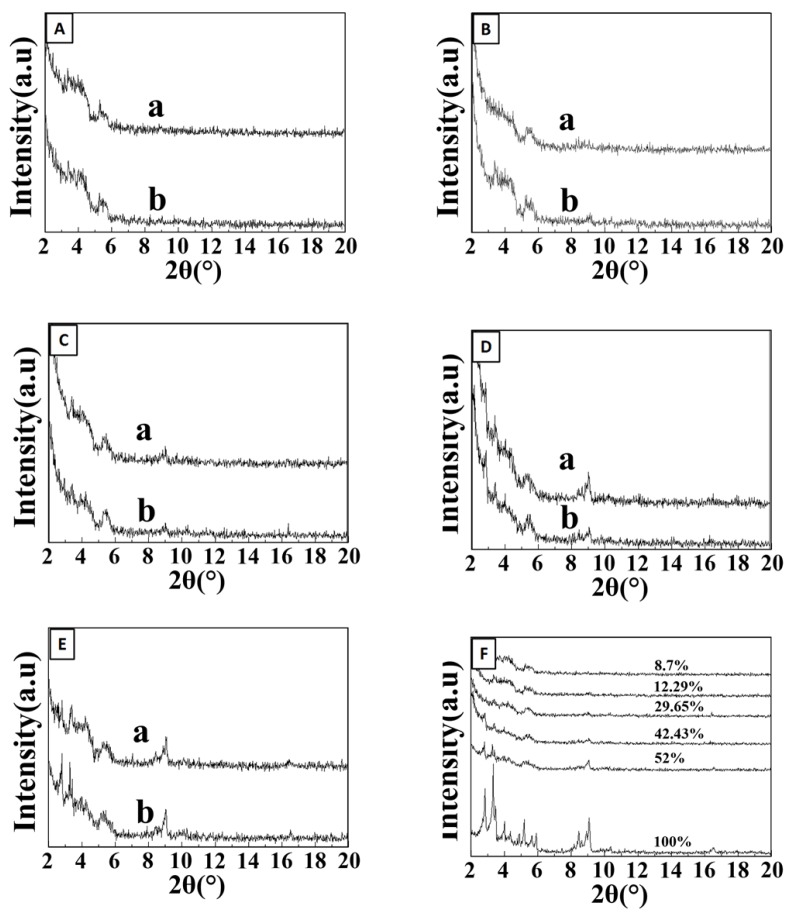
PXRD patterns of MIL-101(Cr) (n%)-SiO_2_ composites (a) before and (b) after water adsorption treatment: (**A**) MIL-101(Cr) (8.70%)-SiO_2_, (**B**) MIL-101(Cr) (12.29%)-SiO_2_, (**C**) MIL-101(Cr) (29.60%)-SiO_2_, (**D**) MIL-101(Cr)(42.43%)-SiO_2_, (**E**) MIL-101(Cr) (52.00%)-SiO_2_, (**F**) different percentages of MIL-101(Cr) in MIL-101(Cr) (n%)-SiO_2_ composites.

Figure 2A–E displays the XRD patterns of the different percentage of MIL-101(Cr)-SiO_2_ composites before and after the water vapor adsorption treatment. As can be seen, the MIL-101(Cr) (0.00%)-SiO_2_, MIL-101(Cr) (8.70%)-SiO_2_, MIL-101(Cr) (12.29%)-SiO_2_, MIL-101(Cr) (29.65%)-SiO_2_, MIL-101(Cr) (42.43%)-SiO_2_ and MIL-101(Cr) (52.00%)-SiO_2_ showed diffraction peaks at 2.83°, 3.31°, 5.18°, 8.45° and 9.07°, respectively. The results indicate that the crystalline structure of the MIL-101(Cr) did not change after the water vapor adsorption treatment. Hence, the MIL-101(Cr)-SiO_2_ composites could be reused for another experiment without further treatment. Figure 2F shows the diffraction peaks at 2.83°, 3.31°, 4.01°, 5.18°, 5.86°, 8.45°, 9.07°, 10.33°, 11.23°, 16.53°, 16.89°, 17.27° and 19.6° confirming the formation of the metal organic framework [41]. The XRD intensity of the MIL-101(Cr) 8.70%-SiO_2_ samples was very weak compared to those samples prepared with a higher percentage of MIL-101(Cr), because of their poor crystallinity. The poor crystalline nature of these composites arose from the smaller percentage of MIL-101(Cr) and higher concentration of silica. At 12.29%, slight 2θ peaks appeared due to the initiation of crystallization. At percentages above 12.29%, the intensity increased more when compared to percentages of 8.70 and 12.29. Based on the results, we can conclude that diffraction peaks began to appear when the amount of added MIL-101(Cr) exceeded 12.26%, with high intensity observed at small angles (2θ), which indicates the presence of mesopores in the MIL-101(Cr) (29.60%)-SiO_2_, MIL-101(Cr) (42.43%)-SiO_2_ and MIL-101(Cr) (52%)-SiO_2_ structures. With a higher composition of MIL-101(Cr) i.e., 29.60%, 42.43% or 52%, the structure was sufficiently well developed to make this a potentially good adsorbent for adsorption chillers.

### 2.3. Microstructural and Compositional Studies

**Figure 3:**
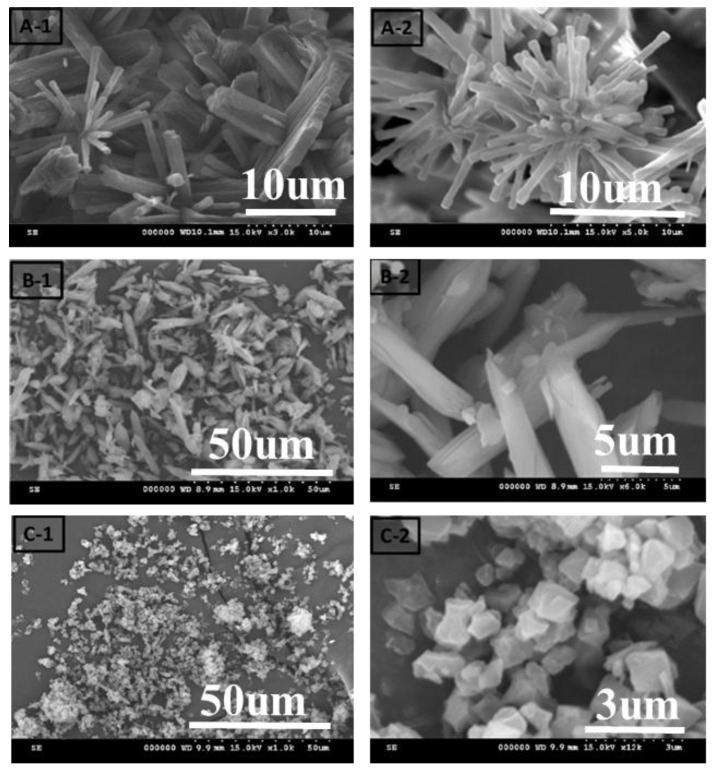
The SEM images of different ratios of chromium nitrate/terephthalic acid/hydrofluoric acid ((**A-1** and **A-2**) 1:1:4, (**B-1** and **B-2**) 1:1:2, and (**C-1** and **C-2**) 1:1:1.4).

**Figure 4:**
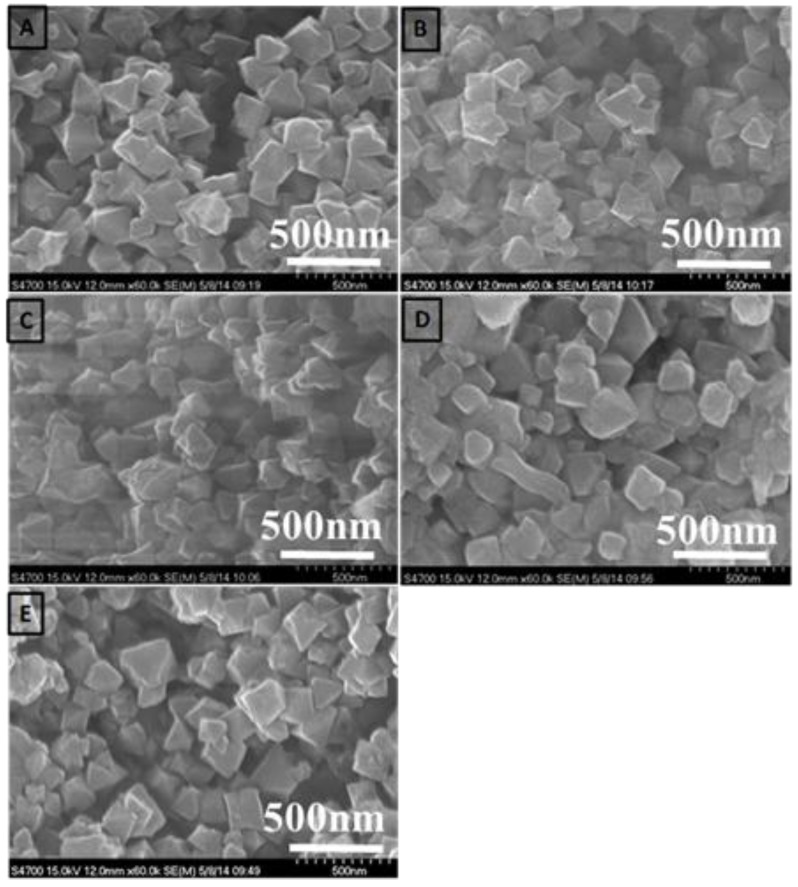
The SEM images of MOF composites prepared using microwave-assisted hydrothermal method with different time interval. (**A**) 20 (**B**) 30 (**C**) 40 (**D**) 50 and (**E**) 60 min.

The SEM images of different ratios of chromium nitrate/terephthalic acid/hydrofluoric acid (1:1:4, 1:1:2, and 1:1:1.4) are shown in Figure 3. When the ratio of hydrofluoric acid decreased from 4 to 1.4 the shape of the MIL-101(Cr) composites changed from a needle to a square shape, and the particle sizes were gradually decreased from 10 to 1 μm. Figure 4 shows the SEM images of MIL-101(Cr) composites prepared using microwave-assisted, hydrothermal method with different time intervals. There is not much difference in the shape of MIL-101(Cr) composites for all the samples with different time intervals, and their sizes are about 300~400 nm as shown in Figure 4.

**Figure 5:**
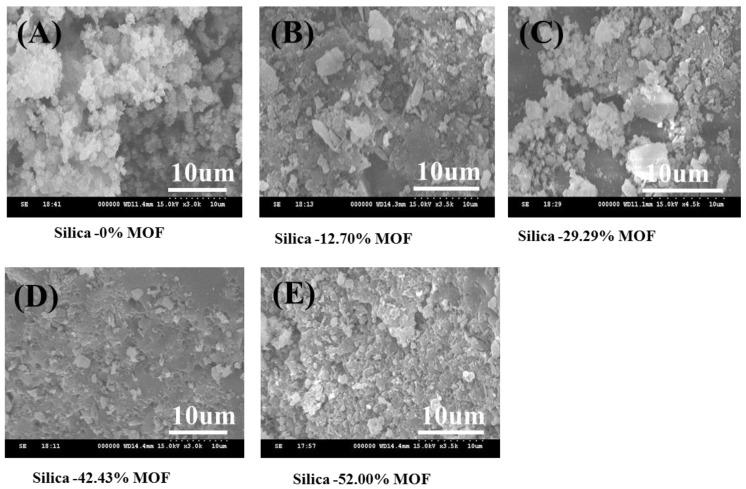
SEM images of the (**A**) MIL-101(Cr) (0.00%)-SiO_2_, (**B**) MIL-101(Cr) (12.29%)-SiO_2_, (**C**) MIL-101(Cr) (29.65%)-SiO_2_, (**D**) MIL-101(Cr) (42.43%)-SiO_2_ and (**E**) MIL-101(Cr) MIL-101(Cr) (52.00%)-SiO_2_.

Further, the SEM was carried out to analyze the microstructure of the MIL-101(Cr) (n%)-Si composites. Figure 5 shows SEM images of (A) MIL-101(Cr) (0.00%)-SiO_2_, (B) MIL-101(Cr) (12.29%)-SiO_2_, (C) MIL-101(Cr) (29.65%)-SiO_2_, (D) MIL-101(Cr) (42.43%)-SiO_2_ and (E) MIL-101(Cr) (52.00%)-SiO_2_ at 2.5K (X). The different concentrations of MIL-101(Cr) in the MIL-101(Cr)-SiO_2_ composites were monodispersed with particle sizes of around 1 μm. Moreover, the morphology of the MIL-101(Cr) (0.00%)-SiO_2_, and MIL-101(Cr) (8.70%)-SiO_2_ differed from that of the MIL-101(Cr) (29.65%)-SiO_2_, MIL-101(Cr) (42.43%)-SiO_2_ and MIL-101(Cr) (52.00%)-SiO_2_ samples. A very low concentration of MIL-101(Cr) loading did not affect the morphology of the MIL-101(Cr)-SiO_2_ composites, whereas at higher loadings such as MIL-101(Cr) (29.65)-SiO_2_, MIL-101(Cr) (42.43%)-SiO_2_, and MIL-101(Cr) (52%)-SiO_2_, there was a change in the surface morphology of the aggregated crystals, which had an irregular granular shape, as shown in Figure 5C–E. Referring to the XRD results, we can observe diffraction peaks for the MIL-101(Cr) (29.65%)-SiO_2_, MIL-101(Cr) (42.43%)-SiO_2_ and MIL-101(Cr) (52%)-SiO_2_ composites, due to the greater aggregation of MIL-101(Cr) with Si composites. The surface composition of all the samples was investigated using energy dispersive X-ray analysis. The elemental surface compositions of all the samples (a–g) are listed in Table 1. The atomic ratios of [Si]:[O]:[Cr] for these MIL-101(Cr) composite particles were in the range of 1.00:2.51–2.98:0.00–0.19. The homogeneity of the dispersion of the Cr, O and silica particles in the MIL-101(Cr) (n%)-Si composite was based on the percentage of MIL-101(Cr). The EDX results agree well with the XRD results, with the MIL-101(Cr) (29.65%)-SiO_2_, MIL-101(Cr) (42.63)-SiO_2_, and (MIL-101(Cr) (52%)-SiO_2_ samples showing a better crystalline phase due to the higher Cr/Si ratio.

### 2.4. Thermogravimetric Analysis (TGA)

**Figure 6:**
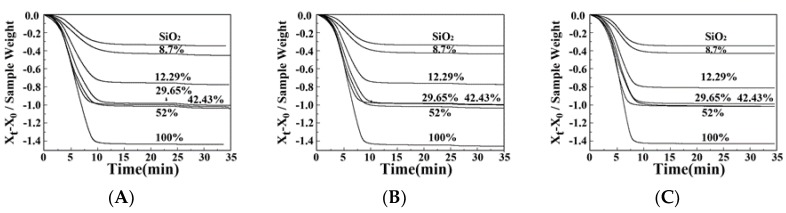
TGA thermograms of MIL-101(Cr) (n%)-SiO_2_ composites under nitrogen atmosphere at (**A**) 75 °C, (**B**) 80 °C, and (**C**) 95 °C.

TGA is one of the best methods for quantitatively estimating the water adsorption of a MIL-101(Cr)-SiO_2_ composite [42], therefore this method was used to observe the thermal stability of the MIL-101(Cr)-SiO_2_ with various temperature ramp rates, as shown in Figure 6. A significant weight loss occurred and a stable zone formed under the nitrogen atmosphere at temperatures of 75, 80 and 95 °C as shown in Figure 6A–C, respectively. In the first stage, there is a weight loss of all samples in the interval from 0 to 15 min, which indicates the completion of dehydration within 15 min. The dehydration time decreased with increasing temperature. In addition, it can be found that the dehydration time decreased with variation of the percentage of MIL-101(Cr) from 0% to 100.00%. After 15 min, the curves of all samples tended to stabilize, due to the completion of dehydration. The TGA results demonstrate that the addition of the MIL-101(Cr) does not affect thermal stability. Abid et al. [41] reported that MIL-101(Cr) has a strong thermal stability, which agrees well with the observations of our MIL-101(Cr)-SiO_2_ samples.

### 2.5. Nitrogen Adsorption Analysis

**Figure 7:**
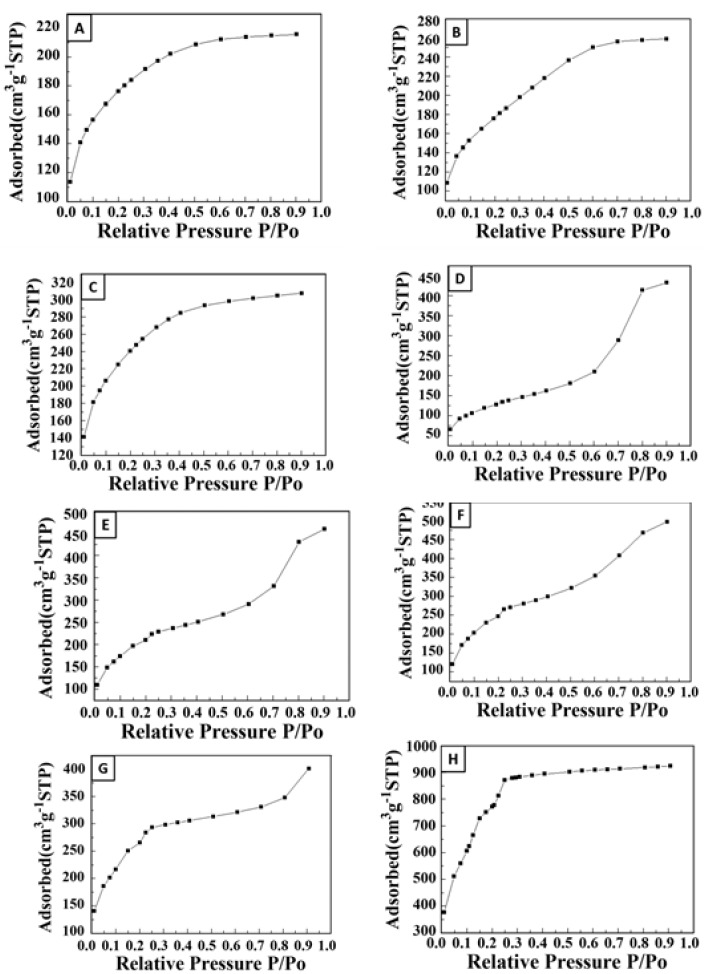
Nitrogen adsorption isotherms for MIL-101(Cr) (n%)-SiO_2_ composites: (**A**) commercial Silica gel, (**B**) MIL-101(Cr) (0%)-SiO_2_, (**C**) MIL-101(Cr) (8.70%)-SiO_2_, (**D**) MIL-101(Cr) (12.29%)-SiO_2_, (**E**) MIL-101(Cr) (29.6)-SiO_2_, (**F**) MIL-101(Cr) (42.43%)-SiO_2_, (**G**) MIL-101(Cr) (52%)-SiO_2_, (**H**) MIL-101(Cr) (100%).

The nitrogen adsorption isotherms for the MIL-101(Cr)-SiO_2_ composite materials are shown in Figure 7. Figure 7A,F shows the commercial silica gel and pristine MOF. The commercial silica gel, pure SiO_2_ and MIL-101(Cr) (8.70%)-SiO_2_ are associated with type I isotherms, according to the IUPAC classification [43,44] which is characteristic of a uniform microporous structure, as shown in Figure 7C. In addition, the hydroxyl functional groups present in the MIL-101(Cr)-Si that lead to an increase in the affinity between water and MIL-101(Cr)-SiO_2_, resulting in higher N_2_ uptake values, increased with increasing MIL-101(Cr) percentages. Although the existence of these functional groups does affect the isotherm values, this effect appears to be marginal at enhancing the affinity of the adsorbent surface to the water molecule [45]. However, the MIL-101(Cr) (12.29%)-SiO_2_, MIL-101(Cr) (29.60)-SiO_2_, MIL-101(Cr) (42.43%)-SiO_2_ and MIL-101(Cr) (52%)-SiO_2_ composite samples showed the type IV isotherms that correspond to a mesoporous material. The N_2_ type IV adsorption isotherm of MIL-101(Cr)-SiO_2_ indicates an increase in the N_2_ uptake values. The results confirm that MIL-10(Cr)-SiO_2_ can be considered an integrated material with excellent adsorption behavior, arising from the high percentage of MIL-101(Cr) and the mesoporous structure. The nitrogen adsorption isotherm types I and IV results agree well with past reported results [46,47]. The BET and Langmuir surface areas of MIL-101(Cr)-SiO_2_ composites with different percentages of MIL-101(Cr) are listed in Table 2. It can be seen that there is an increase in the BET surface area and Langmuir surface area of all the samples with higher percentages of MIL-101(Cr). Moreover, the MIL-101(Cr) (0%)-SiO_2_ sample showed a low surface area (161.56 m^2^/g) due to the mesoporous SiO_2_ with its occluded surfactant. The MIL-101(Cr) (52%) composite had a higher surface area i.e., 381.41 m^2^/g. Qiu et al. and Yan et al. [48,49] reported that MOF had a diluting effect when combined with SiO_2_ which could lead to the formation of mesoporous wormhole structures in the prepared MIL-101(Cr) composites. Our results show that a high adsorption capacity material could be prepared by using a higher percentage of MIL-101(Cr) in the MIL-101(Cr)-SiO_2_ composite, without losing the nature of the mesoporous structure. 

### 2.6. Water Vapor Adsorption Behaviors of MIL-101(Cr)-SiO_2_ Composites

**Figure 8:**
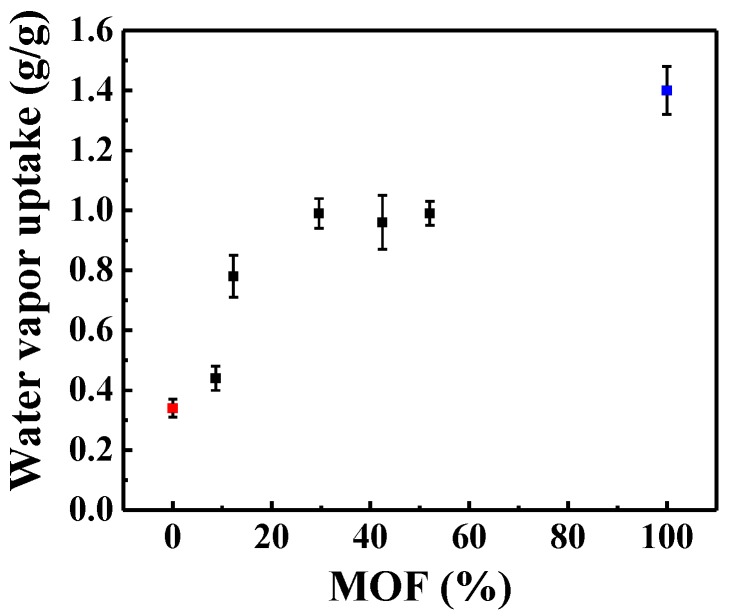
Water vapor adsorption behavior of MIL-101(Cr) (n%)-SiO_2_ composites. Red symbol MIL-101(Cr) (0%)-SiO_2_ and blue symbol MIL-101(Cr) (100%) composites.

The water adsorption isotherms of the MIL-101(Cr) (n%)-SiO_2_ composites are shown in Figure 8. Recent water vapor adsorption results provide significant information for surface characterization of the hydrophilicity/hydrophobicity of MOFs [46,49]. Kim et al. [50] and Pan et al. [36] reported that Lewis sites and Brønsted sites play a dominant role in the water vapor adsorption capacity, being beneficial to the adsorption kinetics on the material surface. In Figure 8, it can be seen that the water vapor absorbance increases being 0.34 (18.88 mM/g), 0.44 (24.44 mM/g), 0.78 (43.33 mM/g), 0.96 (53.3 mM/g), 0.99 (55.0 mM/g), 1.03 (57.22 mM/g), and 1.40 g/g (77.77 mM/g) for the MIL-101(Cr) (0%)-SiO_2_ , MIL-101(Cr) (8.7%)-SiO_2_, MIL-101(Cr) (12.29%)-SiO_2_, MIL-101(Cr) (29.60%)-SiO_2_, MIL-101(Cr) (42.43%)-SiO_2_, MIL-101(Cr) (52%)-SiO_2_ and pure MIL-101(Cr), respectively. The greater number of micropores in the MIL-101(Cr)-SiO_2_ composites to be filled with water, and the pore size of the MIL-101(Cr) structure, play primary roles in increasing the water vapor adsorption capacity. In addition, the Lewis and Brønsted sites gave the strongest intensity in the hydroxyl functional groups on the surface of the materials. This result was also confirmed by the FTIR results, as the Lewis and Brønsted acid increased with a rise in the percentage of MIL-101(Cr) in the composites. Therefore, the MIL-101(Cr)-SiO_2_ composites showed a higher water vapor adsorption capacity and stronger thermal stability due to the high BET and Langmuir surface area, the mesoporous structure, and the Lewis and Brønsted acid sites. We can thus conclude that the MIL-101(Cr)-SiO_2_ composites with more than 12.29% MIL-101(Cr) would have greater water-adsorption ability whereas those with less than 12.29% MIL-101(Cr) embedded in the SiO_2_ matrix would have a restricted water vapor adsorption capacity.

### 2.7. Water Vapor Adsorption and Desorption Stability Test

**Figure 9:**
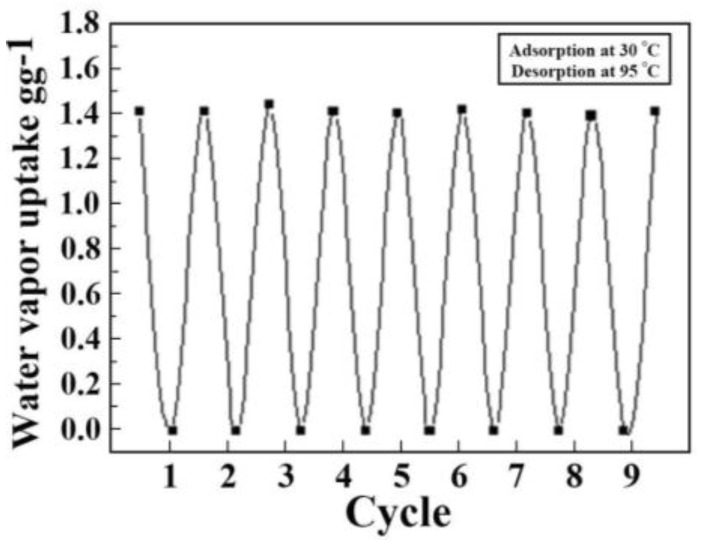
Water vapor adsorption and desorption stability of MIL-101(Cr) (42.43%)-SiO_2_ composites.

The stability of the MIL-101(Cr) (42.43%)-SiO_2_ composites were tested by the adsorptions/desorption (30 °C/95 °C) of water vapor up to eight cycles, as shown in Figure 9. For all the eight cycles, there is no change in the adsorption/desorption capability of the MIL-101(Cr) (42.43%)-SiO_2_ composites in the presence of water vapor. The results confirm that the MIL-101(Cr)-SiO_2_ composites prepared using a microwave-assisted hydrothermal method is more stable and possesses the potential of practical application.

## 3. Material Preparation and Characterization

### 3.1. Chemical Reagents

Tetraethyl orthosilicate (Si (OC_2_H_5_)_4_, 99%), chromium (III) nitrate nonahydrate (Cr (NO_3_)_3_·9H_2_O, 99%), 1,4-benzene dicarboxylic acid (C_12_H_10_O_4_, 99%), hydrofluoric acid (HF, 48%), ammonium fluoride (NH_4_F, 99%), ethanol (CH_3_CH_2_OH, 99%), hydrogen chloride (HCl, 99%) were received from Sigma-Aldrich and used without further purification.

### 3.2. Preparation of MIL-101(Cr)-SiO_2_ Composites

MIL-101(Cr)-SiO_2_ composites with varying MIL-101(Cr) percentages were prepared using the microwave- assisted hydrothermal and sol-gel methods. Briefly the Cr (NO_3_)_3_·9H_2_O (15 mmol), C_12_H_10_O_4_ (23 mmol) and HF (8 mmol) were dissolved in distilled water (15 mL) and continuously stirred to form a homogeneous solution. In this experiment, we tried three different ratios of chromium nitrate, 1,4-benzene dicarboxylic acid and hydrofluoric acid i.e., 1:1:4, 1:1:2 and 1: 1:1.4. The adsorbents were prepared using a microwave-assisted hydrothermal method with a microwave power of 500 W at times of 20, 30, 40, 50 and 60 min. The ratio of 1:1:1.4 and microwave power of 500 W at 60 min were used to prepare MIL-101(Cr). For crystallization, this homogeneous solution was transferred into a Teflon-lined autoclave. The autoclave in a microwave oven was heated to 220 °C for eight h and then allowed to cool down to room temperature. After the treatment, the suspension was filtered and washed with ethanol and ultra-pure water three times, and then dispersed in 1M NH_4_F. After rinsing with ultra-pure water, the as-prepared samples were dried at 100 °C overnight. Subsequently, a sol-gel solution of Si (OC_2_H_5_)_4_ (1.3 mol) and HCl (13 mL) was introduced to the above mixture, and then stirred continuously until gelation was complete. After this, the product was washed with ethanol and water three times. The final product was then dried at 100 °C for 12 h. The samples prepared with various weight percentages of MIL-101(Cr) are denoted by MIL-101(Cr) (n%) (*n* = 0.00, 8.90, 12.29, 29.65, 42.43, 52.00)-SiO_2_. In addition, we prepared pure MIL-101(Cr) samples for comparison purposes.

### 3.3. Characterization of the Samples

In this study, different techniques were used to investigate the physico-chemical properties of the MIL-101(Cr)-SiO_2_ composites. The crystallography of the MIL-101(Cr)-SiO_2_ composites was investigated by Powder X-ray diffraction (XRD, Analytical X’Pert PRO) using filtered CuKα radiation (λ = 1.5418 Å) at a voltage of 45 kV, current of 40 mA, and scanned at 2θ from 2° to 20°. The surface morphology and composition were studied using scanning electron microscopy (SEM, HITACHIS-3000H) with energy-dispersive X-ray analysis (EDX, KEPL JSAM 6700). The specific surface area was measured with N_2_ adsorption isotherms by the Brunauer–Emmett–Teller (BET*, Micromeritics’* *Gemini V*) method. In addition, the thermal stability of all samples was analyzed by thermo gravimetric analysis (TGA, Pyris 6 TGA), under an N_2_ atmosphere. The water vapor adsorption capacity was studied using a Quantachrome Instrument (ChemBET^®^3000 TPR/TPD).

### 3.4. Water Vapor Adsorption Kinetics

Water vapor adsorption tests were carried out in a quartz tube containing 300 mg of each sample using the Quantachrome Instrument. All the samples were dehydrated under a helium flow of 50 mL/min at 120 ± 1 °C for five h before water vapor adsorption analysis. Then the water vapor MIL-101(Cr) (0.1 volume percentage balance with helium) was introduced into the reactor tube at a temperature of 30 ± 1 °C for 150 min. The amount of water vapor MIL-101(Cr) was calculated as the difference between the total and dry weights of the material. This measurement was repeated at least three times in order to confirm the reproducibility of the MIL-101(Cr) kinetics and the average results calculated. In recent years, several research groups have studied thermal characterization using the following kinetics equations [51,52]:

X _water–sorption_ = X_t_ − X_0_/X_t_, (1)

where X_0_ and X_t_ are the total and dry weights of the MIL-101(Cr) composites, respectively.

## 4. Conclusions

A series of MIL-101(Cr)-SiO_2_ composites was successfully synthesized by the microwave-assisted hydrothermal technique, as well as the sol-gel approach. The optimal concentration of 29.6% to 52% of MIL-101(Cr) in the silica was found to produce a well-developed mesoporous structure with a high surface area, which showed a remarkable water vapor adsorption capability. The Lewis and Brønsted sites confirm their porosity, and help to improve the adsorption kinetics on the surface of the material. The results obtained from this work open the door to using the MIL-101(Cr)-SiO_2_ composites in industrial applications in adsorption chillers. 

## Figures and Tables

**Table 1:** Molar ratios of elements present on the MIL-101(Cr) (n%)-SiO_2_ composites.

Sample	Molar Ratios of Elements		
	Si	O	Cr
(a) MIL-101(Cr) (0%)-SiO_2_	1.00	2.98	0.00
(b) MIL-101(Cr) (8.70%)-SiO_2_	1.00	2.87	0.03
(c) MIL-101(Cr) (12.29%)-SiO_2_	1.00	2.73	0.04
(d) MIL-101(Cr) (29.60%)-SiO_2_	1.00	2.65	0.08
(e) MIL-101(Cr) (42.43%)-SiO_2_	1.00	2.63	0.12
(f) MIL-101(Cr) (52%)-SiO_2_	1.00	2.52	0.15
(g) MIL-101(Cr) (100%)	0	2.51	0.19

**Table 2:** BET surface area and Langmuir surface area of MIL-101(Cr) (n%)-SiO_2_ composites.

Sample	BET Surface Area (m^2^/g)	Langmuir Surface Area (m^2^/g)
Commercial SiO_2_	162	981
(a) MIL-101(Cr) (0%)-SiO_2_	193	1221
(b) MIL-101(Cr) (8.70%)-SiO_2_	235	1408
(c) MIL-101(Cr) (12.29%)-SiO_2_	279	2028
(d) MIL-101(Cr) (29.60%)-SiO_2_	344	2143
(e) MIL-101(Cr) (42.43%)-SiO_2_	350	2248
(f) MIL-101(Cr) (52%)-SiO_2_	381	2678
(g) MIL-101(Cr) (100%)	725	4238

## References

[1] Canivet J., Fateeva A., Guo Y., Coasne B., Farrusseng D. (2014). Water adsorption in MOFs: Fundamentals and applications. Chem. Soc. Rev..

[2] Tuan A.V., Le Giang H., Canh D.D., Lan Q.D., Kien T.N., Phuong T.D., Hoa T.D., Tran T.K., Duong Q.T., Nguyen T.V. (2014). Isomorphous substitution of Cr by Fe in MIL-101 framework and its application as a novel heterogeneous photo-Fenton catalyst for reactive dye degradation. RSC Adv..

[3] Li Z., Zeng H.C. (2014). Armored MOFs: Enforcing soft microporous MOF nanocrystals with hard mesoporous silica. J. Am. Chem. Soc..

[4] Jacoby Q.L.M. (2014). New Sorbents for Greener Cooling. Chem. Eng. News.

[5] Hu Z., Xu Q. (2014). Metal–organic framework composites. Chem. Soc. Rev..

[6] Martin W., Annika H., Rene T., Barbara M., Christoph J. (2015). Hierarchical MOF-xerogel monolithic composites from embedding MIL-100 (Fe, Cr) and MIL-101(Cr) in resorcinol-formaldehyde xerogels for water adsorption applications. Microporous Mesoporous Mater..

[7] Ferey G. (2008). Hybrid porous solids: Past, present, future. Chem. Soc. Rev..

[8] Dario B., Kate M., Gimona N.M., Hill J.A., Falcaro P. (2011). Fast Synthesis of MOF-5 Microcrystals Using Sol−Gel SiO_2_ Nanoparticles. Chem. Mater..

[9] Choia J.-S., Sona W.-J., Kimb J., Ahna W.-S. (2008). Metal–organic framework MOF-5 prepared by microwave heating: Factors to be considered. Microporous Mesoporous Mater..

[10] Yaghi O., Li H. (1995). Hydrothermal synthesis of a metal-organic framework containing large rectangular 309 channels. J. Am. Chem. Soc..

[11] Ahmed A., Forster M., Jin J., Myers P., Zhang H. (2015). Tuning morphology of nanostructured ZIF-8 on silica microspheres and applications in liquid chromatography and dye degradation. ACS Appl. Mater. Interfaces.

[12] Song Y., Hu D., Liu F., Chen S., Wang L. (2015). Fabrication of fluorescent SiO_2_@zeolitic imidazolate framework-8 nanosensor for Cu^2+^ detection. Analyst.

[13] Yan Z., Zheng J., Chen J., Tong P., Lu M., Lin Z., Zhang L. (2014). Preparation and evaluation of silica-UiO-66 composite as liquid chromatographic stationary phase for fast and efficient separation. J. Chromatogr. A.

[14] Ameloot R., Liekens A., Alaerts L., Maes M., Galarneau A., Coq B., Desmet G., Sels B.F., Denayer J.F.M., de Vos D.E. (2010). Silica-MOF composites as a stationary phase in liquid chromatography. Eur. J. Inorg. Chem..

[15] Kong S., Dai R., Li H., Sun W., Wang Y. (2015). Microwave Hydrothermal Synthesis of Ni-based Metal–Organic Frameworks and Their Derived Yolk–Shell NiO for Li-Ion Storage and Supported Ammonia Borane for Hydrogen Desorption. ACS Sustain. Chem. Eng..

[16] Amo-Ochoa P., Givaja G., Miguel P.J.S., Castillo O., Zamora F. (2007). Microwave assisted hydrothermal synthesis of a novel Cu I-sulfate-pyrazine MOF. Inorg. Chem. Commun..

[17] Johanna L., Kelly E., Anderson A., Samantha G. (2015). Conway, Hydrothermal Synthesis and Characterization of a Metal–Organic Framework by Thermogravimetric Analysis, Powder X-ray Diffraction, and Infrared Spectroscopy, An Integrative Inorganic Chemistry Experiment. J. Chem. Educ..

[18] Liang Y., Yuan W.G., Zhang S.F., He Z., Xue J., Zhang X., Jing L.H., Qin D.B. (2016). Hydrothermal synthesis and structural characterization of metal–organic frameworks based on new tetradentate ligands. Dalton Trans..

[19] Al-Kutubi H., Gascon J., Sudhölter E.J.R., Rassaei L. (2015). Electrosynthesis of Metal–Organic Frameworks: Challenges and Opportunities. ChemElectroChem.

[20] Yang H.-M., Liu X., Song X.-L., Yang T.-L., Liang Z.-H., Fan C.-M. (2015). In situ electrochemical synthesis of MOF-5 and its application in improving photocatalytic activity of BiOBr. Trans. Nonferr. Met. Soc. China.

[21] Son W.-J., Kim J., Kim J., Ahn W.-S. (2008). Sonochemical synthesis of MOF-5. Chem. Commun..

[22] Abedi S., Tehrani A.A., Morsali A. (2015). Mechanochemical synthesis of isoreticular metal–organic frameworks and comparative study of their potential for nitrobenzene sensing. New J. Chem..

[23] Friscic T. (2014). Metal-Organic Frameworks: Mechanochemical Synthesis Strategies, Encyclopedia of Inorganic and Bioinorganic Chemistry.

[24] Klimakow M. (2010). Mechanochemical synthesis of metal—Organic frameworks: A fast and facile approach toward quantitative yields and high specific surface area. Chem. Mater..

[25] Chen W.-X., Tan L., Liu Q.-P., Qiang G.-R., Zhuang G.-L. (2014). The ionothermal synthesis, structure, and magnetism–structure relationship of two biphenyl tetracarboxylic acid-based metal–organic frameworks. Dalton Trans..

[26] Klimakow M., Klobes P., Thünemann A.F., Rademann K., Emmerling F. (2016). Ionothermal synthesis and proton-conductive properties of NH 2-MIL-53 MOF nanomaterials. CrystEngComm.

[27] Xu L., Choi E.Y., Kwon Y.U. (2008). Ionothermal synthesis of a 3D Zn–BTC metal-organic framework with distorted tetranuclear [Zn 4 (μ 4-O)] subunits. Inorg. Chem. Commun..

[28] Parnham E.R., Morris R.E. (2007). Ionothermal synthesis of zeolites, metal-organic frameworks, and inorganic-organic hybrids. Acc. Chem. Res..

[29] Das A.K., Vemuri R.S., Kutnyakov I., Peter McGrail B., Motkuri R.K. (2016). An Efficient Synthesis Strategy for Metal-Organic Frameworks: Dry-Gel Synthesis of MOF-74 Framework with High Yield and Improved Performance. Sci. Rep..

[30] Ahmed I., Jeon J., Khan N., Jhung S.H. (2012). Synthesis of a Metal–Organic Framework, Iron-Benezenetricarboxylate, from Dry Gels in the Absence of Acid and Salt. Cryst. Growth Des..

[31] Faustini M., Kim J., Jeong G.Y., Kim J.Y., Moon H.R., Ahn W.S., Kim D.P. (2013). Microfluidic approach toward continuous and ultrafast synthesis of metal–organic framework crystals and hetero structures in confined microdroplets. J. Am. Chem. Soc..

[32] Liang K., Ricco R., Reboul J., Furukawa S., Falcaro P., Levy D., Zayat M. (2015). Sol–Gel for Metal Organic Frameworks (MOFs). The Sol-Gel Handbook-Synthesis, Characterization, and Applications: Synthesis, Characterization and Applications.

[33] Furukawa H., Gándara F., Zhang Y., Wendy J.J., Queen L., Matthew R., Hudson R., Omar M., Yaghi M. (2014). Water adsorption in porous metal-organic frameworks and related materials. J. Am. Chem. Soc..

[34] Burtch N.C., Jasuja H., Walton K.S. (2014). Water stability and adsorption in metal-organic frameworks. Chem. Rev..

[35] Sienkiewicz-Gromiuk J., Rusinek I., Kurach Ł., Rzączyńska Z. (2006). Thermal and spectroscopic (IR, XPS) properties of lanthanide (III) benzene-1,3,5-triacetate complexes. J. Therm. Anal. Calorim..

[36] Pan G.-T., Lai M.-H., Juang R.-C., Chung T.-W., Yang T.C.-K. (2011). Preparation of visible-light-driven silver vanadates by a microwave-assisted hydrothermal method for the photodegradation of volatile organic vapors. Ind. Eng. Chem. Res..

[37] Brown D.W., Floyd A.J., Sainsbury M. (1988). Organic Spectroscopy.

[38] Pan G.-T., Chong S., Yang T.C.-K., Yang Y.-L., Arjun N. (2016). Surface Modification of Amorphous SiO_2_ Nanoparticles by Oxygen-Plasma and Nitrogen-Plasma Treatments. Chem. Eng. Commun..

[39] Fazaeli R., Aliyan H., Moghadam M., Masoudinia M. (2013). Nano-rod catalysts: Building MOF bottles (MIL-101 family as heterogeneous single-site catalysts) around vanadium oxide ships. J. Mol. Catal. A Chem..

[40] Yang J., Zhao Q., Li J., Dong J. (2010). Synthesis of metal–organic framework MIL-101 in TMAOH-Cr (NO 3) 3-H 2 BDC-H 2 O and its hydrogen- storage behavior. Microporous Mesoporous Mater..

[41] Abid H.R., Ang H.M., Wang S. (2012). Effects of ammonium hydroxide on the structure and gas adsorption of nanosized Zr-MOFs (UiO-66). Nanoscale.

[42] Wang S., Bromberg L., Schreuder-Gibson H., Alan Hatton T. (2013). Organophophorous Ester Degradation by Chromium (III) Terephthalate Metal−Organic Framework (MIL-101) Chelated to N,N-Dimethylaminopyridine and Related Aminopyridines. Appl. Mater. Interfaces.

[43] Donohue M., Aranovich G. (1998). Classification of Gibbs adsorption isotherms. Adv. Colloid Interface Sci..

[44] Neves G.M., Lenza R.F.S., Vasconcelos W.L. (2002). Evaluation of the Microwaves in the Structure of Silica Gels. Mater. Res..

[45] Rong X.Z., Du H.K., Wang S.-X., Liu D.-W. (2006). Chang, A.-H. Effects of taurine on type I and III collagen expression in rats lung exposed to silica. Chin. J. Ind. Hyg. Occup. Dis..

[46] Mu B., Walton K.S. (2011). Thermal Analysis and Heat Capacity Study of Metal–Organic Frameworks. J. Phys. Chem. C.

[47] Yan X., Hu X., Komarneni S. (2014). Facile synthesis of mesoporous MOF/silica composites. RSC Adv..

[48] Qiu L.-G., Xu T., Li Z.-Q., Wang W., Wu Y., Jiang X., Tian X.-Y., Zhang L.-D. (2008). Hierarchically Micro-and Mesoporous Metal–Organic Frameworks with Tunable Porosity. Angew. Chem. Int. Ed..

[49] Yang D.-A., Cho H.-Y., Kim J., Yang S.-T., Ahn W.-S. (2012). CO_2_ capture and conversion using Mg-MOF-74 prepared by a sonochemical method. Energy Environ. Sci..

[50] Kim S.N., Yang S.T., Kim J., Park J., Ahn W.-S. (2012). Post-synthesis functionalization of MIL-101 using diethylenetriamine: A study on adsorption and catalysis. CrystEngComm.

[51] Tsung-Yu T., Wu P.-C., Liao K.-T., Huang H.-Y., Lin C.-H., Hsu J.-S., Lee W. (2015). Purification of deteriorated liquid crystals by employing porous metal–organic-framework/polymer composites. Opt. Mater. Exp..

[52] Chong S., Pan G.-T., Khalid M., Yang T.C.-K., Hung S.-T., Huang C.-M. (2016). Physical Characterization and Pre-assessment of Recycled High-Density Polyethylene as 3D Printing Material. J. Polym. Environ..

